# Plant Species Recovery on a Compacted Skid Road

**DOI:** 10.3390/s8053123

**Published:** 2008-05-15

**Authors:** Murat Demir, Ender Makineci, Beyza Sat Gungor

**Affiliations:** 1 Istanbul University, Faculty of Forestry, Forest Construction and Transportation Department 34473 Bahcekoy / Sariyer / Istanbul / Turkey; E-mail: mdemir@istanbul.edu.tr; 2 Istanbul University, Faculty of Forestry, Soil Science and Ecology Department 34473 Bahcekoy / Sariyer / Istanbul / Turkey; E-mail: emak@istanbul.edu.tr; 3 Istanbul University, Faculty of Forestry, Landscape Architecture Department 34473 Bahcekoy / Sariyer / Istanbul / Turkey; E-mail: beyza@istanbul.edu.tr

**Keywords:** Timber skidding, timber harvesting, harvesting effects, soil disturbance, logging, herbaceous plant species

## Abstract

This study was executed to determine the plant species of herbaceous cover in a skid road subjected to soil compaction due to timber skidding in a beech (*Fagus orientalis* Lipsky.) stand. Our previous studies have shown that ground based timber skidding destroys the soils extremely, and degradations on ecosystem because of the timber skidding limit recovery and growth of plant cover on skid roads. However, some plant species show healthy habitat, recovery and they can survive after the extreme degradation in study area. We evaluated composition of these plant species and their cover-abundance scales in 100 m x 3 m transect. 15 plant species were determined belongs to 12 plant families and Liliaceae was the highest representative plant family*. Smilax aspera* L., *Epimedium pubigerum* (DC.) Moren et Decaisne, *Carex distachya* Desf. var. *distachya* Desf.*, Pteridium aquilinum* (L.) Kuhn., *Trachystemon orientalis* (L.) G. Don, *Hedera helix* L. have the highest cover-abundance scale overall of determined species on compacted skid road.

## 1. Introduction

Roads are critical components of civilization. Developing and maintaining the economic activity that is vital for the quality of modern life would be difficult without roads. Roads provide access for people to study, enjoy, or contemplate natural ecosystems [1-3]. Building and maintaining roads have become controversial, however, because of public concerns about their short and long term effects on the environment [4]. Potential ecological impacts of roads have received a great deal of attention in recent years. Particularly prominent management issues and research problems include effects of roads on soil erosion, hydrology and aquatic ecosystems. Effects of roads on forest plant communities are less well-documented [5]. Logging operations in forests can cause significant and wide spread soil disturbance, including removal, mixing and compaction of various soil layers. Disturbance can adversely affect both soil physical properties and soil nutrient levels to such an extent that severely diminished growth of subsequent tree rotations as well as significant increase in runoff and sediment loads may result [6]. That the production works being carried out in the forest have many negative impacts on the forest ecosystem is very well known. It has also been determined that the production and skidding negatively affect the amounts and variety of forest floor and herbaceous understory as well as youth development and living conditions of the soil organisms [7-15]. Skidding or yarding on terrain requires the construction of relatively dense network of forest roads including skid roads, haul roads and landings [16]. Skid roads are defined as tertiary roads that are used by skidders and forwarders that move logs from the point of felling and bucking to log landings. Apart from the felling of occasional trees to provide a clear path, few improvements are made to skid roads [5]. The dimension of the impact created by skidding of the products directly on the ground varies according to many factors such as slope, site characteristics, production methods used, planning of skid roads and production season. Ground-based skidding may result in soil compaction and other soil structural changes, influencing soil water retention, and reducing soil aeration, drainage, and root penetration. Studies focused on exotic plant invasions in roadside plant communities along main forest and grassland roads and in adjacent communities have been completed, but changes in plant composition within interior haul roads and skid trails have received little attention. The extent of haul road and skid road networks in managed forests and linkages between understory vascular plant communities and other flora and fauna, the impacts of these features on ecosystem properties and processes could be substantial [5].

In this study, we evaluated survived herbaceous plant species composition and their cover-abundance scales on the skid road subjected to compaction due to ground based timber skidding activities that have been carried out for many years (since 1956) in a beech (*Fagus orientalis* Lipsky.) stand.

## 2. Materials and Methods

Research area was in the boundaries of zone 82 of Istanbul Belgrad forest. Belgrad forest covering a surface area of 5441.71 ha located in the Marmara geographical region between latitudes 41° 09′ - 41° 12′ N and longitudes 28° 54′ - 29° 00′ E. According to the data given by Bahcekoy meteorology station, average annual precipitation is 1074.4 mm, average annual temperature is 12.8°C, average maximum temperature is 17.8°C and the average minimum temperature is 9°C. The climate of Istanbul Belgrad Forest is close to sea (ocean) climate with medium water deficit in summers. Vegetation period maintains for 7.5 months (230 days) in average. The period of study is October 2006 and research area is oriental beech (*Fagus orientalis* Lipsky) stand having canopy cover as 0.8, average diameter as 23.12 cm (the height of breast height above ground level is 1.30 m), average height as 24.14 m and stand density as 1400 trees/ha. Average altitude of the research area is 140 m, slope is 10-15% with SW aspect. The skidding road passing through the stand in west-east direction has long been used (since 1956) to skid the logs out of the area. It was estimated that 135 m^3^ timbers was skidded annually in harvest activities on the skid road. Last using date of skid road is in October 2004. Skid road width is 3.0 m [17]. The harvested timbers are also being towed by tractors with a rope along with skidding by means of manpower and animals.

We evaluated impacts of disturbance in interior skid road on understory herbaceous vegetation by documenting the areal extent of these features and plant composition along 100 m x 3 m belt transect in October 2006. During sampling 100m tapes were streched along transect to delineate skid road boundaries. Transect was oriented accros the road, perpendicular to its main axis (as described in [5]). The species composition on the transect on skid road was characterized by classical phytosociological plots according to Braun-Blanquet cover-abundance values [18]. which means that total coverage for each species (vertical projection onto the ground) was estimated visually and recorded within seven cover classes: r: 1 or 5 individuals; +: few individuals (<20) with cover <5%; 1: many individuals (20– 100) with cover <5%; 2: 5%–25% cover; 3: 25%–50% cover; 4: 50%–75% cover; 5: 75%–100% cover [14, 18]. Easily identified herbaceous plant species on skid road reported on the field, undetermined ones clipped and brought to the laboratory to determine by comparing the samples in Istanbul University Faculty of Forestry Herbarium and guide books. The general site characteristics of herbaceous plant species were reported as described in [19].

## 2. Results and Discussion

The skidding works that have been carried on for many years in beech (*Fagus orientalis* Lipsky) stand caused decreasing of the forest floor and the herbaceous cover on the skid road to a great extent. Furthermore, there were some other important changes in the soil properties at the two examined soil depths (0-5 cm and 5-10 cm) (Table 1). The major impact that occurred down to 10 cm soil depth was the compaction of soil. Higher volume and fine soil weight values compared to those of the undisturbed area were found at both the depths on the skid road subject to the soil compaction. The total porosity and moisture equivalence on the skid road decreased considerably [20]. In addition there were no significant differences for chemical properties of soil at 0-5 cm soil depth (N, P, K, Na, Ca, Mg, Fe, Zn, Cu and Mn). A significant difference between soil samples at 5-10 cm depth, taken from skid road and undisturbed area was found with respect to Mg (143.85 ppm) concentrations [21]. These previous results clearly show that skidding has particularly negative impacts on herbaceous cover, organic layer and soil. These effects of skidding on skid road have been demonstrated to have detrimental impacts on native flora, and herbaceous plant establishing and maintaining were limited. Similarly, Mariani et al. (2006) [22] reported that organic layer removal reduced abundance of herbs and shrubs. Soil compaction can also severely reduce plant growth by restricting root growth may be due to oxygen stress and lower the percentage of water and air space in the soil [23]. Also, Kozlowski (1999) [24] mentioned a reduced total photosynthesis when soils become increasingly compacted, as a result of smaller leaf areas.

On the other hand, likely more adapted (especially to soil compaction) and less herbaceous plant mass exist on skid road. We evaluated these plant species and their cover-abundance scales in 100m × 3 m transect. According to results obtained from this study, 15 plant species were determined belongs to 12 plant family (Table 2). Liliaceae was the highest representative plant family (Table 2). *Smilax aspera* L., *Epimedium pubigerum* (DC.) Moren et Decaisne, *Carex distachya* Desf. var. *distachya* Desf.*, Pteridium aquilinum* (L.) Kuhn., *Trachystemon orientalis* (L.) G. Don, *Hedera helix* L. have the highest cover-abundance scale overall of determined species on compacted skid road (Table 2). 4 herbaceous plant species have second cover abundance scale (+, few, with small cover) on the skid road including; *Lamium purpureum* L. var. *purpureum* L.*, Ruscus hypoglossum* L., *Geranium asphodeloides* Burm. Fil. subsp. *asphodeloides* Burm. Fil., *Rubus discolor* Weihe et Nees (Table 2). And other plant species including *Stellaria holostea* L., *Primula vulgaris* Huds. subsp. *vulgaris* Huds., *Fragaria vesca* L., *Doronicum orientale* Hoffm., *Ornithogalum wiedemannii* Boiss., have the “r” cover abundance scale, and they were solitary with small cover on the skid road (Table 2). Obtaining results show that determined plant species have the ability to grow on extreme soil and site conditions. Some earlier studies in other regions have also proved the survival capability most of these species on degraded, polluted or disturbed habitats which limit plant growth and survival as summarized on Table 3.

Effects of skid roads on soil properties and other site factors have been demonstrated to have detrimental impacts on native flora and fauna. And the capacity of vegetation recover depends on the ability of individual species to recover, habitat factors such as soil properties, microclimate and associate species composition. Establishing and maintaining native plant communities on forest roads is vital because until adequate plant cover is established, ripping alone provides only temporary and marginal improvements in soil structure. Herbaceous plant cover on roads can improve soil structure by increasing soil organic matter, reducing erosion and restoring the habitat lost. Successful revegetation may also help to keep roads closed by reducing their visibility. For these reasons, determining and availability of revegetation, recovery and survival success of native plant species have particular importance. Identification of native species would be successful to rehabilitate skid roads. In conclusion, the determined herbaceous species on skid road in this study show the resistance and survival capability on degraded soil and micro environment conditions.

## Figures and Tables

**Table 1.** Investigated soil properties in soil on skid road [20,21].

Characteristics	Units	0-5 cm	5-10 cm
Sand	(%)	58.05	51.35
Silt	(%)	20.74	30.88
Clay	(%)	21.21	17.77
Soil Acidity	pH	5.49	5.07
Electrical Conductivity	(μhos cm^-1^)	81.32	48.02
Fine soil (<2mm) weight	(g cm^–3^)	0.763	906.55
Coarse soil (>2mm) weight	(g cm^–3^)	0.137	147.15
Root mass	(g cm^–3^)	2.76	2.79
Organic Carbon	(%)	10.20	7.32
Moisture Equivalent	(%)	24.37	24.44
Total Porosity	(%)	52.72	50.22
Moisture	(%)	20.86	18.73
Compaction	(kg cm^-2^)	2.17	2.69
Bulk Density	(g cm^–3^)	0.902	1.09
N	(%)	0.358	0.198
P	(ppm)	3.83	1.08
K	(ppm)	105.41	54.72
Na	(ppm)	19.21	15.90
Ca	(ppm)	1706.44	579.13
Mg	(ppm)	263.52	143.85
Fe	(ppm)	1.11	0.74
Zn	(ppm)	112.71	37.86
Cu	(ppm)	0.86	1.19
Mn	(ppm)	250.83	188.72

**Table 2.** Survived plant species on compacted skid road, their general site characteristics and the cover-abundance scales.

The Plant Family	The Scientific Name of the Plant Species	General Site Characteristics	The Cover-Abundance Scale
CARYOPHYLLACEAE	*Stellaria holostea* L.	Scrubs, roadsides, damp places.	r
PRIMULACEAE	*Primula vulgaris* Huds. subsp. *vulgaris* Huds.	Often damp places in open or shady turfslopes, evergreen or deciduous woodlands, alpine meadows.	r
LILIACEAE	*Smilax aspera* L.	Macchie, scrub, ravines, and rocky limestone slopes.	1
BERBERIDACEAE	*Epimedium pubigerum* (DC.) Moren et Decaisne	Forest clearings	1
LABIATAE	*Lamium purpureum* L. var. *purpureum* L.	Oak and fir forests, gravelly banks, fields and waste places.	+
LILIACEAE	*Ruscus hypoglossum* L.	Mixed forests, scrubs, ravines, rocky places.	+
ROSACEAE	*Fragaria vesca* L.	Moist places, especially in forest.	r
CYPERACEAE	*Carex distachya* Desf. var. *distachya* Desf.	Dry stony slopes, open forests, roadsides	1
GERANIACEAE	*Geranium asphodeloides* Burm. Fil. subsp. *asphodeloides* Burm. Fil.	Forests, scrubs, meadows, banks.	+
COMPOSITAE	*Doronicum orientale* Hoffm	Shady paces in forests and scrubs..	r
LILIACEAE	*Ornithogalum wiedemannii* Boiss	Woods and forests	r
DENNSTAEDTIACEAE	*Pteridium aquilinum* (L.) Kuhn.	In forest clearings, cleared woodland, dunes.	1
BORAGINACEAE	*Trachystemon orientalis* (L.) G. Don	Fir forests, shady riverbanks, moist ravines.	1
ARALIACEAE	*Hedera helix* L.	Climbing over trees or creeping on the ground in woods.	1
ROSACEAE	*Rubus discolor* Weihe et Nees.	Deciduous forests and scrubs, shady banks, coastal plains.	+

**Table 3.** The characteristics and place of disturbed-degraded habitat where the plants can growth.

Plant Species	The characteristics and place of disturbed-degraded habitat	References
*Stellaria holostea* L.	under atmospheric nitrogen deposition, N deposited oak forest, Denmark	[25]
	oak dune forest, Denmark	[26]
	recovered on footpaths that have been closed for access for 6 years, Belgium	[27]
	under disturbed *Q. pyrenaica* ecosystems by human intervention, Spain	[28]
	under wooded landscapes human influented, UK	[29]

*Primula vulgaris* Huds. subsp. *vulgaris* Huds.	restoration of wet grasslands, Germany	[30]

*Smilax aspera* L.	undergrowth clearings of the coppice oak forests, Spain	[31]
	disturbed forest areas, potential restoration of natural vegetation, Spain	[32]
	forest disturbances (grazing and thinning), Spain	[33]

*Epimedium pubigerum* (DC.) Moren et Decaisne	-	-

*Lamium purpureum* L. var. *purpureum* L.	radioactive-contaminated sites around nuclear power plant, Slovak Republic	[34]
	under the Mediterranean dryland conditions, effects of tillage systems, Turkey	[35]
	man made habitats with irregular disturbances, Czech Republic	[36]

*Ruscus hypoglossum* L.	in a nature park, Croatia	[37]

*Fragaria vesca* L.	oak dune forest, Denmark	[26]
	restoration of heathland/moorland, UK	[38]
	response to cattle grazing in mesic semi-natural grassland, Finland	[39]
	restoration success in alluvial grasslands under contrasting flooding regimes, Germany	[40]
	norway spruce forests following clearcutting and shelterwood cutting, Sweden	[41]
	contaminated soil of uranium waste depot, Czech Republic	[42]

*Carex distachya* Desf. var. *distachya* Desf.	plant reestablishment 15 years after the debris avalanche, USA- described as *Carex* sp.	[43]
	restoration of heathland/moorland, UK- described as *Carex* sp.	[38]
	forests following clearcutting and shelterwood cutting, Sweden. described as *Carex* sp.	[41]

*Geranium asphodeloides* Burm. Fil. subsp. *asphodeloides* Burm. Fil.	around villages in Sakarya province, Turkey	[44]

*Doronicum orientale* Hoffm.	on basaltic pyroclastic deposits at different altitudes, Italy	[45]
	on mountainous area (Yunt Mountain), Turkey	[46]

*Hedera helix* L.	recovered on footpaths that have been closed for access for 6 years, Belgium	[27]
	undergrowth clearings of the coppice oak forests, Spain	[31]
	forest disturbances (grazing and thinning), Spain	[33]
	pine forest clearings along the French Atlantic sand dunes, France	[47]
	in reclaimed area contaminated with dioxin, Italy	[48]
	man made urban habitats, urban-industrial ecosystems, Germany	[49]

*Rubus discolor* Weihe et Nees.	forest disturbances (grazing and thinning), Spain-described as Rubus sp.	[33]

*Ornithogalum wiedemannii* Boiss.	-	-

*Pteridium aquilinum* (L.) Kuhn.	Initial recovery after wildfire and clear cut in Jack pine forest, USA	[50]
	pine forest clearings along the French Atlantic sand dunes, France	[47]
	under disturbed *Q. pyrenaica* ecosystems by human intervention, Spain	[28]
	glades, rides and roads in plantation forests, Ireland	[51]
	Short-term understory plant community responses to timber-harvesting intensity, USA	[52]

*Trachystemon orientalis* (L.) G. Don	after woody vegetation control treatments (bulldozing and hand-grubbing), Turkey	[53]
	around villages in Sakarya province, Turkey	[44]

## References

[1] Crisholm M. (1990). The increasing separation of production and consumption. The Earth as Transformed by Human Action.

[2] Grübler A. (1994). Technology. Changes in land use and land cover: a global perspective.

[3] Demir M. (2007). Impacts, management and functional planning criterion of forest road network system in Turkey. Transportation Research Part A: Policy and Practice.

[4] Cole D.N., Landres P.B. (1996). Threats to wilderness ecosystems: impacts and research needs. Ecological Applications.

[5] Buckley D.S., Crow T.R., Nauertz E.A., Schulz K.E. (2003). Influence of skid trails and haul roads on understory plant richness and composition in managed forest landscapes in Upper Michigan, USA. Forest Ecology and Management.

[6] Laffan M., Jordan G., Duhig N. (2001). Impacts on soils from cable-logging steep slopes in Northeastern Tasmania, Australia. Forest Ecology and Management.

[7] Messina M.G., Schoenholtz S.H., Lowe M.W., Wang Z., Gunter D.K., Londo A.J. (1997). Initial responses of woody vegetation, water quality, and soils to harvesting intensity in a texas bottomland hardwood ecosystem. Forest Ecology and Management.

[8] Wang L. (1997). Assessment of animal skidding and ground machine skidding under mountain conditions. Journal of Forest Engineering.

[9] Bengtsson J., Lundkvist H., Saetre P., Sohlenius B., Solbreck B. (1998). Effects of organic matter removal on the soil food web: forestry practices meet ecological theory. Applied Soil Ecology.

[10] Arocena J.M. (2000). Cations in solution from forest soils subjected to forest floor removal and compaction treatments. Forest Ecology and Management.

[11] Marshall V.G. (2000). Impacts of forest harvesting on biological processes in Northern forest soils. Forest Ecology and Management.

[12] Gilliam F.S. (2002). Effects of harvesting on herbaceous layer diversity of a central appalachian hardwood forest in West Virginia, USA. Forest Ecology and Management.

[13] Williamson J.R., Neilsen W.A. (2003). The effect of soil compaction, profile disturbance and fertilizer application on the growth of eucalypt seedlings in two glasshouse studies. Soil and Tillage Research.

[14] Godefroid S., Koedam N. (2004). The impact of forest paths upon adjacent vegetation: effects of the paths surfacing material on the species composition and soil compaction. Biological Conservation.

[15] Johnston F.M., Johnston S.W. (2004). Impacts of road disturbance on soil properties and exotic plant occurrence in subalpine areas of Australian Alps. Arctic Antarctic and Alpine Research.

[16] Ketcheson G.L., Megahan W.F., King J.G. (1999). “R1-R4” and “Boised” sediment prediction model tests using forest roads in granitics. Journal of American Water Resource Assessment.

[17] Anonymous (2005). Harvesting reports of Istanbul-Bahcekoy Regional Directorate of Forestry.

[18] Braun-Blanquet J. (1964). Pflanzensoziologie-Grundzüge der Vegetationskunde..

[19] Davis P.H. Flora of Turkey and East Aegean Islands.

[20] Demir M., Makineci E., Yilmaz E. (2007). Harvesting impacts on herbaceous understory, forest floor and top soil properties on skid road in a beech (*Fagus orientalis* Lipsky.) stand. Journal of Environmental Biology.

[21] Demir M., Makineci E., Comez A., Yilmaz E. (2008). Timber harvesting effects on chemical properties of topsoil, herbaceous cover and forest floor in a beech (*Fagus orientalis* Lipsky.) stand. Journal for Nature Conservation.

[22] Mariani L., Chang S.X., Kabzems R. (2006). Effects of tree harvesting, forest floor removal, and compaction on soil microbial biomass, microbial respiration, and N availability in boreal aspen forest in British Coloumbia. Soil Biology and Biochemistry.

[23] Barzegar A.R., Nadian H., Heidari F., Herbert S.J., Hashemi A.M. (2006). Interaction of soil compaction, phosphorus and zinc on clover growth and accumulation of phosphorus. Soil and Tillage Research.

[24] Kozlowski T.T. (1999). Soil compaction and growth of woody plants. Scandinavian Journal of Forest Research.

[25] Kristensen H.L., Henriksen K. (1998). Soil nitrogen transformations along a successional gradient from *Calluna* heathland to Q*uercus* forest at intermediate atmospheric nitrogen deposition. Applied Soil Ecology.

[26] Lawesson J.E., Wind P. (2002). Oak dune forests in Denmark and their ecology. Forest Ecology and Management.

[27] Roovers P., Bossuyt B., Gulinck H., Hermy M. (2005). Vegetation recovery on closed paths in temperate deciduous forests. Journal of Environmental Management.

[28] Tárrega R., Calvo L., Marcos E., Taboada A. (2007). Comparison of understory plant community composition and soil characteristics in *Quercus pyrenaica* stands with different human uses. Forest Ecology and Management.

[29] Rotherham R.I. (2007). The implications of perceptions and cultural knowledge loss for the management of wooded landscapes: A UK case-study. Forest Ecology and Management.

[30] Rosenthal G. (2003). Selecting target species to evaluate the success of wet grassland restoration. Agriculture, Ecosystems and Environment.

[31] Camprodon J., Brotons L. (2006). Effects of undergrowth clearing on the bird communities of the northwestern Mediterranean coppice holm oak forests. Forest Ecology and Management.

[32] Arévalo J.R., Fernández-Palacios J.M. (2005). Gradient analysis of exotic *Pinus radiata* plantations and potential restoration of natural vegetation in Tenerife, Canary Islands (Spain). Acta Oecologica.

[33] Onaindia M., Dominguez I., Albizu I., Garbisu C., Amezaga I. (2004). Vegetation diversity and vertical structure as indicators of forest disturbance. Forest Ecology and Management.

[34] Mičieta K., Murín G. (2007). Wild plant species in bio-indication of radioactive-contaminated sites around Jaslovske Bohunice nuclear power plant in the Slovak Republic. Journal of Environmental Radioactivity.

[35] Ozpinar S. (2006). Effects of tillage systems on weed population and economics for winter wheat production under the Mediterranean dryland conditions. Soil and Tillage Research.

[36] Lososová Z., Chytrý M., Kühn I., Hájek O., Horáková V., Pyšek P., Tichý L. (2006). Patterns of plant traits in annual vegetation of man-made habitats in central Europe. Perspectives in Plant Ecology, Evolution and Systematics.

[37] Jelaska S.D., Antonić O., Nikolić T., Hršak V., Plazibat M., Križan J. (2003). Estimating plant species occurrence in MTB/64 quadrants as a function of DEM-based variables—a case study for Medvednica Nature Park, Croatia. Ecological Modelling.

[38] Tong C., Le Duca M.G., Ghorbani J., Mars R.H. (2006). Linking restoration to the wider landscape: A study of a bracken control experiment within a upland moorland landscape mosaic in the Peak District, UK. Journal of Landscape Urban Planning.

[39] Pykälä J. (2005). Plant species responses to cattle grazing in mesic semi-natural grassland. Agriculture, Ecosystems and Environment.

[40] Bissels S., Hölzel N., Donath T.W., Otte A. (2004). Evaluation of restoration success in alluvial grasslands under contrasting flooding regimes. Biological Conservation.

[41] Hannerz M., Hånell B. (1997). Effects on the flora in Norway spruce forests following clearcutting and shelterwood cutting. Forest Ecology and Management.

[42] Soudek P., Petřík P., Vágner M., Tykva R., Václav P., Petrová S., Vaněk T. (2007). Botanical survey and screening of plant species which accumulate ^226^Ra from contaminated soil of uranium waste depot. European Journal of Soil Biology.

[43] Dale V.H., Adams W.M. (2003). Plant reestablishment 15 years after the debris avalanche at Mount St.Helens, Washington. Science of the Total Environment.

[44] Uzun E., Sariyar G., Adsersen A., Karakoc B., Otuk G., Oktayoglu E., Pirildar S. (2004). Traditional medicine in Sakarya province (Turkey) and antimicrobial activities of selected species. Journal of Ethnopharmacology.

[45] Fernández Sanjurjo M.J., Corti G., Certini G., Ugolini F.C. (2003). Pedogenesis induced by Genista aetnensis (Biv.) DC. on basaltic pyroclastic deposits at different altitudes, Mt. Etna, Italy. Geoderma.

[46] Ugurlu E., Secmen O. (2007). Medicinal plants popularly used in the villages of Yunt Mountain (Manisa-Turkey). Fitoterapia.

[47] Lemauviel S., Roze F. (2000). Ecological study of pine forest clearings along the French Atlantic sand dunes: Perspectives of restoration. Acta Oecologica.

[48] Sartori F., Assini S. (2001). Vegetation evolution in reclaimed area contaminated with dioxin. Chemosphere.

[49] Bornkamm R. (2007). Spontaneous development of urban woody vegetation on differing soils. Flora.

[50] Le Duc S.D., Rothstein D.E. (2007). Initial recovery of soil carbon and nitrogen pools and dynamics following disturbance in jack pine forests: A comparison of wildfire and clearcut harvesting. Soil Biology and Biochemistry.

[51] Smith G.F., Iremonger S., Kelly D.L., O'donoghue S., Mitchell F.J.G. (2007). Enhancing vegetation diversity in glades, rides and roads in plantation forests. Biological Conservation.

[52] Fredericksen T.S., Ross B.D., Hoffman W., Morrison M.L., Beyea J., Johnson B.N., Lester M.B., Ross E. (1999). Short-term understory plant community responses to timber-harvesting intensity on non-industrial private forestlands in Pennsylvania. Forest Ecology and Management.

[53] Yildiz O., Sarginci M., Esen D., Cromack K. (2007). Effects of vegetation control on nutrient removal and Fagus orientalis, Lipsky regeneration in the western Black Sea Region of Turkey. Forest Ecology and Management.

